# Supplementary material for: Cervical cancer screening utilization and associated factors among female health workers in public health facilities of Hossana town, southern Ethiopia: A mixed method approach
Source: PLoS One. 2023 May 30;18(5):e0286262. doi: 10.1371/journal.pone.0286262 (PMC10228814; doi:10.1371/journal.pone.0286262)
Supplement: S1 Data — (DOCX) [file pone.0286262.s001.docx]

**Annex II: English version Questionnaires**

Cervical cancer screening utilization and associated factors among female health workers in public health facilities of Hosanna town, Southern Ethiopia

Name of the Health Facility: _____________________________________

Questionnaire Identification Number: ________________

*** N.B: you can select more than one answer that are mentioned below**

**Part A: Socio-demographic characteristics of the respondents**

| **S/No** | **Questions** | **Response and Coding** | **Skip to Q** |
| --- | --- | --- | --- |
| **A1** | Age (years) | ____________years |  |
| **A2** | Ethnicity | 1. Hadiya 2. Kembata 3. Gurage 4. Siltie 5. Amhara 6. Others (specify)____________ |  |
| **A3** | Religion | 1. Protestant 2. Muslim 3. Orthodox 4. Catholic 5. Others (specify)____________ |  |
| **A4** | Current marital status | 1. Married 2. Single 3. Divorced 4. Widowed |  |
| **A5** | What is your parity? | 1. Nullipara 2. 1-2 child 3. 3-4 child 4. >5 child |  |
| **A6** | Where is your duty of station? | _______________________________. |  |
| **A7** | Your level of education? | 1. Diploma 2. 1 st Degree 3. Post graduate 4. 4. Others (specify)................ |  |
| **A8** | Your profession? | 1. Nurse 2. Health Officer 3. Doctors 4. Pharmacy 5. Lab. Technician/technologist 6. Midwife 7. Others (Specify)............... |  |
| **A9** | Your service year duration? | ………………………… in years |  |

**Part B. Knowledge on cervical cancer and screening of precancerous cervical lesion**

| **B1** | where did you get information about cervical cancer and screening | | | 1. Print Media 2. Electronic media 3. Training 4. Campaign 5. Friends 6. Doctor 7. Others (specify)..................... |  | |
| --- | --- | --- | --- | --- | --- | --- |
| B2 | Do you know the cervical cancer screening service is available in your resident/ health facility | | 1. Yes 2. No | | |  |
| B3 | What are the risk factors for cervical cancer? | | 1. Having multiple sexual partners 2. Early sexual intercourse 3. Acquiring HPV virus 4. Cigarette smoking 5. Don’t know | | |  |
| B4 | Who is vulnerable to cervical cancer? | | 1. women more than 50 years old 2. reproductive age women 3. Both 4. Don’t know | | |  |
| B5 | How can a person prevent getting cancer of the cervix? | | 1. Avoid multiple sexual partners’ 2. Avoid early sexual intercourse 3. Quit cigarette smoking 4. HPV vaccination 5. Do not know | | |  |
| B6 | What are the ways of cervical cancer screening? | 1. Pap Smear 2. VIA (visual inspection with acetic acid) 3. VILI (visual inspection with Lugol’) 4. HPV DNA test 5. Do not know | | | |  |
| B7 | How frequent is screening for premalignant cervical lesion done? | 1. Once every year 2. Once every three years 3. Once every 5 years 4. Do not know 5. Any other mention.......................... | | | |  |
| B9 | Who should be screened? | 1. Women of 30 years and above 2. women age >21 year 3. Elderly women 4. don’t know 5. Others Specify……………….............. | | |  | |
| B10 | What are the symptoms of cervical cancer? | 1. Vaginal bleeding 2. Vaginal foul smelling discharges 3. Contact bleeding 4. Post-menopausal bleeding 5. Do not know | | |  | |

**Part C. Attitude/perception about cervical cancer and screening**

| C1 | Do you believe cervical cancer is one of the most killer cancers in our country? | 1. Strongly agree 2. Agree 3. Neutral 4. Disagree 5. strongly disagree |  |
| --- | --- | --- | --- |
| C2 | Do you believe Screening helps in the prevention of carcinoma of the cervix? | 1. Strongly agree 2. Agree 3. Neutral 4. Disagree 5. strongly disagree |  |
| C3 | Do you believe Screening causes no harm to the client? | 1. 1 Strongly agree 2. Agree 3. Neutral 4. Disagree 5. strongly disagree |  |
| C4 | I will screened for cervical cancer if the service need payment | 1. Strongly agree 2. Agree 3. Neutral 4. Disagree 5. Strongly disagree |  |
| C5 | Do you perceive any adult woman including you can be acquiring cervical carcinoma? | 1. 1 Strongly agree 2. Agree 3. Neutral 4. Disagree 5. strongly disagree |  |
| C6 | Do you believe Screening test can find cervical changes before it become cancer? | 1. 1 Strongly agree 2. Agree 3. Neutral 4. Disagree 5. strongly disagree |  |
| C7 | Do you think to go through screening procedure is embarrassment? | 1. Strongly agree 2. Agree 3. Neutral 4. Disagree 5. strongly disagree |  |
| C8 | If you want to get a screening, will you allow male doctors to examine your cervix? | 1. Strongly agree 2. Agree 3. Neutral 4. Disagree 5. strongly disagree |  |
| C9 | If you develop cervical cancer ,will you consult doctors without being scare | 1. Strongly agree 2. Agree 3. Neutral 4. Disagree 5. strongly disagree |  |

**Part D: Utilization towards screening for premalignant cervical lesion**

|  | | **Questions** | | **Response and Coding** | **Skip to Q** | |
| --- | --- | --- | --- | --- | --- | --- |
| **D1** | | Have you ever screened for cervix cancer | | 1. Yes 2. No | If your answer is ‘’NO’’ go to question D7 | |
| **D2** | | By which method of screening did you screened | | 1. pap smear 2. VIA 3. I don’t know |  | |
| **D3** | | what is your reason to be screened | | 1. To maintain good health 2. To detect early cervical cancer 3. Because of signs and symptoms 4. Since the service is free 5. Other (specify)..................... |  | |
| **D4** | | How many times did you screened? | | 1. Once 2. More than once |  | |
| **D5** | | When was the last time you screened? | | 1. Within the past three years 2. More than three years ago |  | |
| **D6** | | Who recommended you to be screened | | 1. Myself 2. Doctor 3. Nurse 4. Other (specify).............. |  | |
| **D7** | | If your answer is ‘’no’’ for question No 25, why not screened for cervical cancer? | | 1. Fear of positive result 2. Don’t know the place of service 3. Fear of pain 4. Embarrassment 5. I am healthy 6. Carelessness 7. lack of time 8. Other (specify)............... |  | |
| **D8** | | Will you recommend screening to others | | 1. Yes 2. No |  | |
| **D9** | | Why not recommend screening to others | | 1.it is Painful  2.Embarrassing  3. Time consuming  4. Others (specify).................... |  | |
| **D10** | | Is that time of screening convenient? | | 1. Yes 2. No | If your answer is ‘’NO’’ go to question D11 | |
| **D11** | | If your answer is ‘’NO’’ for question D10, which time convenient for you | | 1. For health workers out of governmental working days 2. For health workers out of governmental working hours 3. Preparing campaign for health workers 4. Other (specify ……………. |  | |
| **Part E: Reproductive Health and Behavioural history of respondents** | | | | | | |
| **E1** | At what age your first sexual contact? | | **………year** | | |  |
| **E2** | Do you have multiple sexual partners? | | 1. Yes 2. No | | |  |
| **E3** | Do you have history of sexually transmitted disease? | | 1. Yes 2. No | | |  |
| **E4** | Do you smoke cigarette? | | 1. Yes 2. No | | |  |

|  | | |
| --- | --- | --- |
| **Interview guide for key informants**  Barriers to utilization of cervical cancer screening by female health worker | | |
| Question Numbers | Root questions | Probes |
| Part-I-Socio-demographic information**(** | | |
| 1.1 | How old are you? [-----years]*?* |  |
| 1.2 | Sex (Male) (Female) |  |
| 1.3 | Religion  ______________________ |  |
| 1.4 | Work place |  |
| 1.5 | Educational status |  |
| 1.6 | Profession |  |
| 1.7 | Marital status |  |
| 1.8 | Parity status |  |
| Part-II- Barriers to utilization of cervical cancer and related issues | | |
| 2.1. | How do you characterize female health worker in general regarding cervical cancer screening? | What do they need? Why they are not exposed themselves for screening utilization?  What to do to encourage them? |
| 2.2 | What do you feel about cervical cancer screening utilization? | Has the ability to access utilization changed over time? Has it always been the same or has it been different in the past? How? Why? |
|  |  |  |
| 2.4 | What do you think about the challenges/barriers to utilize cervical cancer screening service? (Focus on facility level, providers’ level, community level and health system level) | what do you feel they are not using the screening services?  How is service delivery to female health worker adversely affected by:  **Health facility** (screening center) ( space, waiting time of services, supplies, convenience,  **Providers** (competency, attitude, discrimination?  **Female health worker** ( KAP, fear, preference, etc)  **Broader health system (**inter-sectoral collaboration, commitment, strategy, policy etc.  **Community** (Families? Peers? Religious fathers? )  What others? |
| 2.4 | What do you feel improves/facilitates to utilizing the services for female health worker? | What makes it easier for them to utilizing the service? |
| 2.5 | What do you do to help female health workers to utilizing cervical cancer screening? | Can you describe when you helped someone to utilizing cervical cancer screening? |
| 2.6 | Are there any other problems you’d like to add or tell me? | ???? |
| Ask throughout the process as appropriate | | |
| 2.6. | What changes do you feel would minimize the impact of  [ Each of the barrier mentioned]? |  |
| 2.7 | What types of programs do you feel would minimize the impact of [Each of the barrier mentioned]? |  |

Thank you !
