# Supplementary material for: Cervical cancer screening utilization and associated factors among female health workers in public health facilities of Hossana town, southern Ethiopia: A mixed method approach
Source: PLoS One. 2023 May 30;18(5):e0286262. doi: 10.1371/journal.pone.0286262 (PMC10228814; doi:10.1371/journal.pone.0286262)
Supplement: Attachment — Submitted filename: PONE-commented.pdf [file pone.0286262.s003.pdf]

PLOS ONE

Cervical cancer screening utilization and associated factors among female health workers in public health facilities of Hosanna town, southern Ethiopia: A mixed method approach

--Manuscript Draft--

Manuscript Number:	PONE-D-22-33914
Article Type:	Research Article
Full Title:	Cervical cancer screening utilization and associated factors among female health workers in public health facilities of Hosanna town, southern Ethiopia: A mixed method approach
Short Title:	Cervical cancer screening utilization and associated factors among health workers in southern Ethiopia
Corresponding Author:	Habtamu Hassen, MSc<br>Hosanna College of Health Inc<br>hosanna, ETHIOPIA
Keywords:	cervical cancer, screening, health worker, Ethiopia
Abstract:	Abstract<br>Backgrounds<br>Worldwide, a substantial portion of women had low cervical cancer screening services utilization. Cervical cancer screening practices among female health workers are paramount in preventing cervical cancer due to health workers' roles to play in promoting the utilization of cervical cancer screening services. However, there is a paucity of evidence in utilization of cervical cancer screening services among female health workers and inconsistent findings in Ethiopia. This study aimed to assess the magnitude of cervical cancer screening utilization and associated factors among female health workers in public health facilities of Hosanna town, Southern Ethiopia.<br>Methods<br>Facility-based cross-sectional study design complemented with the qualitative inquiry was conducted among randomly selected 241 study participants in Hossana town from June 1 to July 1, 2021. Logistic regression models were used to determine the association between dependent and independent variables with the assumption of a variable with a p-value < 0.05 was considered statistically significant. Qualitative data were transcribed verbatim then translated to English and analyzed using open code version 4.03.<br>Results<br>Out of the total study participants, 19.6% were screened for cervical cancer. Having a diploma level of education (AOR=0.48;95%CI:0.24,0.98), having three or more children (AOR=3.65;95%CI:1.44,9.21), having multiple sexual partners(AOR=3.89;95%CI: 1.38,11.01), and knowledge of cervical cancer screening (AOR=2.66;95% CI:1.19,5.95) was statistically significantly associated with cervical cancer screening utilization. In-depth interviews suggested additional barriers for low screening utilization including lack of health educational materials, limitation of service to a specific area, service interruption, provider incompetency, and miss-trust and lack of attention by a trained provider.<br>Conclusion<br>The magnitude of cervical cancer screening service utilization among female health workers is low. Having a diploma level of education, having three or more children, a history of multiple sexual partners, and knowledge about cervical cancer were predictors of cervical cancer screening utilization. Contextualized health talks and promotion through training with a special focus on low level of knowledge, had lower educational level, and the availability of cervical cancer screening services are critical.<br>Keywords: cervical cancer, screening, health worker, Ethiopia
Order of Authors:	Habtamu Hassen, MSc
	Zemzem Jemal, MPH in reproductive Health
	Nana Chea, MPH in reproductive Health

Powered by Editorial Manager® and ProduXion Manager® from Aries Systems Corporation

	Tsegaab Tesfaye, Msc<br>Netsanet Abera, Ass. professor/MPH in reproductive Health
**Additional Information:**	
**Question**	**Response**
**Financial Disclosure**<br><br>Enter a financial disclosure statement that describes the sources of funding for the work included in this submission. Review the submission guidelines for detailed requirements. View published research articles from *PLOS ONE* for specific examples.<br><br>This statement is required for submission and **will appear in the published article** if the submission is accepted. Please make sure it is accurate.<br><br>**Unfunded studies**<br>Enter: *The author(s) received no specific funding for this work.*<br><br>**Funded studies**<br>Enter a statement with the following details:<br>• Initials of the authors who received each award<br>• Grant numbers awarded to each author<br>• The full name of each funder<br>• URL of each funder website<br>• Did the sponsors or funders play any role in the study design, data collection and analysis, decision to publish, or preparation of the manuscript?<br>• **NO** - Include this sentence at the end of your statement: *The funders had no role in study design, data collection and analysis, decision to publish, or preparation of the manuscript.*<br>• **YES** - Specify the role(s) played.<br><br>* typeset	No ,the funders had no role in study design, data collection and analysis, decision to publish, or preparation of the manuscript.
**Competing Interests**<br><br>Use the instructions below to enter a competing interest statement for this submission. On behalf of all authors, disclose any competing interests that could be perceived to bias this work—acknowledging all financial support and any other relevant financial or non-	The authors have declared that no competing interests exist

financial competing interests.<br><br>This statement is required for submission and **will appear in the published article** if the submission is accepted. Please make sure it is accurate and that any funding sources listed in your Funding Information later in the submission form are also declared in your Financial Disclosure statement.<br><br>View published research articles from *PLOS ONE* for specific examples.<br><br>**NO authors have competing interests**<br><br>Enter: *The authors have declared that no competing interests exist.*<br><br>**Authors with competing interests**<br><br>Enter competing interest details beginning with this statement:<br><br>*I have read the journal's policy and the authors of this manuscript have the following competing interests: [insert competing interests here]*<br><br>* typeset	
**Ethics Statement**<br><br>Enter an ethics statement for this submission. This statement is required if the study involved:<br><br>• Human participants<br>• Human specimens or tissue<br>• Vertebrate animals or cephalopods<br>• Vertebrate embryos or tissues<br>• Field research<br><br>Write "N/A" if the submission does not require an ethics statement.<br><br>General guidance is provided below. Consult the submission guidelines for	Hawassa University college of medicine and health sciences institutional review board with approval number of IRB/158/13.<br>written consent were taken from female health workers

detailed instructions. **Make sure that all information entered here is included in the Methods section of the manuscript.**<br><br>**Format for specific study types**<br><br>**Human Subject Research (involving human participants and/or tissue)**<br>• Give the name of the institutional review board or ethics committee that approved the study<br>• Include the approval number and/or a statement indicating approval of this research<br>• Indicate the form of consent obtained (written/oral) or the reason that consent was not obtained (e.g. the data were analyzed anonymously)<br><br>**Animal Research (involving vertebrate animals, embryos or tissues)**<br>• Provide the name of the Institutional Animal Care and Use Committee (IACUC) or other relevant ethics board that reviewed the study protocol, and indicate whether they approved this research or granted a formal waiver of ethical approval<br>• Include an approval number if one was obtained<br>• If the study involved *non-human primates*, add *additional details* about animal welfare and steps taken to ameliorate suffering<br>• If anesthesia, euthanasia, or any kind of animal sacrifice is part of the study, include briefly which substances and/or methods were applied<br><br>**Field Research**<br><br>Include the following details if this study involves the collection of plant, animal, or other materials from a natural setting:<br>• Field permit number<br>• Name of the institution or relevant body that granted permission	
**Data Availability**<br><br>Authors are required to make all data underlying the findings described fully available, without restriction, and from the time of publication. PLOS allows rare	Yes - all data are fully available without restriction

exceptions to address legal and ethical concerns. See the PLOS Data Policy and FAQ for detailed information.<br><br>A Data Availability Statement describing where the data can be found is required at submission. Your answers to this question constitute the Data Availability Statement and **will be published in the article**, if accepted.<br><br>**Important:** Stating 'data available on request from the author' is not sufficient. If your data are only available upon request, select 'No' for the first question and explain your exceptional situation in the text box.<br><br>Do the authors confirm that all data underlying the findings described in their manuscript are fully available without restriction?	
**Describe where the data may be found in full sentences. If you are copying our sample text, replace any instances of XXX with the appropriate details.**<br><br>• If the data are **held or will be held in a public repository**, include URLs, accession numbers or DOIs. If this information will only be available after acceptance, indicate this by ticking the box below. For example: *All XXX files are available from the XXX database (accession number(s) XXX, XXX.)*.<br>• If the data are all contained **within the manuscript and/or Supporting Information files**, enter the following: *All relevant data are within the manuscript and its Supporting Information files.*<br>• If neither of these applies but you are able to provide **details of access elsewhere**, with or without limitations, please do so. For example:<br><br>*Data cannot be shared publicly because of [XXX]. Data are available from the XXX Institutional Data Access / Ethics Committee (contact via XXX) for researchers who meet the criteria for*	All relevant data are within the manuscript and its Supporting Information files.

access to confidential data.<br><br>*The data underlying the results presented in the study are available from (include the name of the third party and contact information or URL).*<br>• This text is appropriate if the data are owned by a third party and authors do not have permission to share the data.<br><br>* typeset	
Additional data availability information:	Tick here if the URLs/accession numbers/DOIs will be available only after acceptance of the manuscript for publication so that we can ensure their inclusion before publication.

Powered by Editorial Manager® and ProduXion Manager® from Aries Systems Corporation

Manuscript

Click here to access/download;Manuscript;Manuscript of cervical cancer.docx

Cervical cancer screening utilization and associated factors among female health workers in public health facilities of Hosanna town, southern Ethiopia: A mixed method approach

Zemzem Jemal[1], Nana Chea[2], Habtamu Hassen[3*], Tsegaab Tesfaye[4], Netsanet Abera[5]

[1] Department of Midwifery, Hossana College of Health Science, Hossana, Ethiopia

[2,5] College of Medicine and Health Sciences, School of Public Health, Hawassa University, Hawassa, Ethiopia

[3 ,4] Department of Public health, Hossana College of Health Science, Hossana, Ethiopia

*Corresponding author,

Email: habtamu130@gmail.com

Abstract

Backgrounds

Worldwide, a substantial portion of women had low cervical cancer screening services utilization. Cervical cancer screening practices among female health workers are paramount in preventing cervical cancer due to health workers' roles to play in promoting the utilization of cervical cancer screening services. However, there is a paucity of evidence in utilization of cervical cancer screening services among female health workers and inconsistent findings in Ethiopia. This study aimed to assess the magnitude of

cervical cancer screening utilization and associated factors among female health workers in public health facilities of Hosanna town, Southern Ethiopia.

Methods

Facility-based cross-sectional study design complemented with the qualitative inquiry was conducted among randomly selected 241 study participants in Hossana town from June 1 to July 1, 2021. Logistic regression models were used to determine the association between dependent and independent variables with the assumption of a variable with a p-value < 0.05 was considered statistically significant. Qualitative data were transcribed verbatim then translated to English and analyzed using open code version 4.03.

Results

Out of the total study participants, 19.6% were screened for cervical cancer. Having a diploma level of education (AOR=0.48;95%CI:0.24,0.98), having three or more children (AOR=3.65;95%CI:1.44,9.21), having multiple sexual partners(AOR=3.89;95%CI: 1.38,11.01), and knowledge of cervical cancer screening (AOR=2.66;95% CI:1.19,5.95) was statistically significantly associated with cervical cancer screening utilization. In-depth interviews suggested additional barriers for low screening utilization including lack of health educational materials, limitation of service to a specific area, service interruption, provider incompetency, and miss-trust and lack of attention by a trained provider.

Conclusion

The magnitude of cervical cancer screening service utilization among female health workers is low. Having a diploma level of education, having three or more children, a history of multiple sexual partners, and knowledge about cervical cancer were predictors of cervical cancer screening utilization. Contextualized health talks and promotion through

training with a special focus on low level of knowledge, had lower educational level, and the availability of cervical cancer screening services are critical.

Keywords: cervical cancer, screening, health worker, Ethiopia

Introduction

Cervical cancer (CC) is the fourth most common cancer detected among women globally and ranks second cancer as the most incident and mortal cancer among women in sub-Saharan Africa [1.2].

There were approximately 236,000 deaths from cervical cancer worldwide and it was the most common cancer in the east and middle Africa [3.4]. About 90% of cases and 85% of these deaths have occurred in Low and Middle-Income Countries (LMICs); the highest has occurred in Sub-Saharan Africa (SSA) and approximately 311,000 women died from cervical cancer [2].

The cause of cervical cancer is Human Papilloma Virus (HPV) and it is the most common viral infection of the reproductive tract, nearly all sexually active individuals will be infected with HPV at some point in their lives and some may repeatedly get infected [3].

Cervical cancer screening is watching for precursors before a person has any symptoms and has the benefit to reduce the incidence and the progression to an advanced stage of cancer as well as its mortality [5].

In Ethiopia, the utilization of cervical cancer screening is low and vary in different setting for instance the prevalence of cervical cancer utilization in Arba Minch town, Southern Ethiopia (9.6%,) Sidama zone Southern Ethiopia(11.4%), and Mekelle town, northern Ethiopia(10.7%) among female health worker [6-8].

The lower rate of cervical cancer screening at low- income countries may be related to the complexity of screening service and the common inherent barriers in the setting such as other socio-cultural issues, limited access to information, lack of knowledge of cervical cancer, lack of healthcare infrastructure required, lack of trained practitioners and the absence of sustained prevention programs [9].

Global commitments to reduce the burden of cervical cancer have been done. For instance, the World Health Organization designed a 90–70-90 triple-intervention strategy aimed to achieve 90% HPV vaccination coverage, 70% of women being screened at least twice in their lifetime, and 90% of women having access to cervical pre-cancer and cervical cancer treatment and palliative care services by 2030 [10].

American obstetrics and gynecology recommended that the initiation of screening with pap smear at the age of 21 every three years interval until the age of 29 years followed by screening with pap smear and HPV testing every five years until the age of 65 [11]. Ethiopia practiced cervical cancer screening services age at least every three years based on WHO recommendations [12].

There is a limited number of studies that have considered factors associated with the female health worker's utilization of cervical cancer screening services in Ethiopia. However, none of these studies have assessed the association between the convenience of screening time and the barrier to utilizing cervical cancer screening services were not qualitatively captured. Therefore, this study aimed to determine the prevalence of cervical cancer screening services utilization and associated factors among female health workers. Health workers are chiefpromotors of health care programs for their community, particularly; female health workers are role models that likely to have a better

understanding of the benefits of cervical screening than others are and their utilization is a predictor of societal health behavior on the control of cervical cancer [13].

Methods and Materials

Study Area

This study was conducted in Hosanna town, the capital town of Hadiya Zone, found in the southern nation nationalities people's regional state (SNNPR) of Ethiopia. The town is situated 232 Km Southwest of Addis Ababa and 194 Km northwest of the regional town of Hawassa. It has an average elevation of 2276 meters above sea level and a total area of 23sq.km. The total population of Hosanna town is 92,735 of this female accounts for 48808 and there are 21,607 reproductive age group women. In Hosanna town administration there is one teaching Hospital, and three health centers namely Hosanna health center, Bobicho health center, and Lichamba health center.

In Hossana town public health facilities there are 643 female health workers such as 247 clinical nurses,113 midwives, 59 laboratory technologists, and technicians,55 pharmacists and druggists, 49 medical doctors, 39 health officers, 13 anesthetics, 37 urban health extension workers, 11 public nurses, six psychiatry nurses, seven environmental nurses, and five radiographers. Screening service for cervical cancer was given in the teaching Hospital and Hosanna health center [1[illegible]

Study design, period, and population

A facility-based cross-sectional study complemented with the qualitative inquiry was conducted from June 1 to July 1, 2021.

The source population comprised all female health workers working in the public health facilities of Hosanna town, while the study population encompassed randomly selected female health workers in selected public health facilities of Hosanna town. Female health workers who were working in selected public health facilities of Hosanna town and whose age is 21 and above were included in this study. Critically ill female health workers who had a total hysterectomy, and were on leave during data collection time were excluded from the study.

Sample size determination and sampling technique

The sample size was calculated by applying two population proportion formulas using Epi-Info version 7 and taking a 5% margin error, 80% power, and a 1:1 ratio of an exposed group to a non-exposed group (r = 1). Assuming the proportion of attitude towards screening of cervical cancer (85.9%) and (AOR=3.42) from a study conducted in Mekelle Town [8]. The calculated sample size was 332.

Since study population was small need correction formula, nf = n/1+ n/N

nf= 332/1+332/643=219

Considering the non-response rate of 10% in the estimation of the sample size required for the study. The final sample size for this study was 241.

A simple random sampling technique was used to recruit study participants. In Hossana town, there are four public health facilities (one hospital & three health centers). The sample size was allocated proportionally based on the number of female health workers. For each health facility, a sampling frame was prepared from the payroll of the human resources department in each public health facility in Hosanna town (Fig 1).

Fig 1: Schematic presentation of the sampling procedure of this study

Data collection tool and procedure

Data were collected using a structured and pretested questionnaire adapted in the English language from the available relevant literature that addresses the objective of the stud [6-8]. The questionnaires contained five parts, Socio-demographic factors, knowledge of cervical cancer and screening-related factors, attitude/perception about cervical cancer and screening, and reproductive health and behavioral factors. The qualitative in-depth interview (IDIs) were conducted using a semi-structured interview guide to gain a deeper understanding of the participants to explore the barriers to utilization of cervical cancer screening. IDIs were administered to a purposely-selected subset of selected female health workers working in the cervical cancer-screening unit, who have management experience in leading the cervical cancer unit, have led the cervical cancer unit of town health administration, and female health workers in Hosanna town public health facility. IDIs were audio-recorded, translated from the local language into English, and then transcribed verbatim.

Operational definitions

Female health workers: Female health professionals who have contact with patients/clients including nurses, doctors, health officers, lab technicians/technologists, pharmacists/ druggists, anesthetics, radiographers, urban health extension workers, public nurses, psychiatry nurses, and environmental nurses [6].

Utilization of cervical screening: whoever screened at least once for cervical cancer [

Knowledge about cervical cancer screening: was assessed using ten questions asked on knowledge of cervical cancer screening (risk factors about cervical cancer, prevention

method, vulnerability to cervical cancer and symptoms of cervical cancer, frequency of screening, and screening method of cervical cancer). The response to each of the questions was “yes” or “no”. Each correct answer was given a score of 1 while an incorrect answer was given a score of 0. We obtained composite knowledge ranging from 0 to 10 points. The scores from all 10 items were summed up and the mean sums of total scores were calculated. A female health worker who obtained scores of the mean and greater than the mean score was considered to have good knowledge and who obtained less than the mean score were considered to have poor knowledge [15].

Attitude/perception of female health workers towards cervical cancer screening was assessed using a likers scale which ranges from score five (strongly agree) to score one (strongly disagree). The responses were summed and a total score was obtained. Then we calculated the mean score. Those who scored the mean score and above were considered as having a favorable attitude or otherwise unfavorable attitudes toward cervical cancer screening [15].

Measurements

The outcome variable, the utilization of cervical cancer screening, was measured through female health worker responses about where she ever screened at least once for cervical cancer.

Data quality assurance

The questionnaires were translated to Amharic and then back-translated to English to assure the quality of data. Three-day training for data collectors and supervisors was given and the questionnaire was pretested in 10% of the study population in a different setting with a similar population in Fonko health center. Cronbach’s alpha was done to

assess internal consistency (alpha coefficient for knowledge on CCA (10 items) = 0.76, attitude on CCA screening (9 items) = 0.71.

Interview guides were prepared in the English language by language experts for qualitative study. Interviews were held in silent places which is suitable and comfortable for discussions. The audio recorder was checked for functionality before recording. During an interview the respondent's own words and crosschecked with notebooks. The recorded voice of interviews and notebooks were crosschecked while transcribing to ensure the credibility of the data.

Data processing and analysis

The quantitative data were entered into Epidata version 3.1 and transported to SPSS version 23 software for analysis. Data were edited and cleaned by running a simple frequency, cross-tabulations, and sorting to identify outliers. Descriptive statistics like frequencies, percentages, and cross-tabulations were done. Binary logistic regression was used to check the associations of independent variables and outcome variables. Variables with p-values < 0.25 in the bivariable analysis were entered into multivariable analysis to isolate predictors. The goodness of fit of the model was checked using the Hosmer Lemeshow test of goodness of fit and variance inflation factors were low (<10) for the multi-collinearity check. An adjusted odds ratio with 95% confidence intervals and a p-value less than 0.05 were considered a statistically significant association with of utilization of cervical cancer screening.

All the qualitative data were systematically coded and analyzed using thematic analysis in open code 4.03 software. The audio recorder was transcribed verbatim in Amharic and then translated into English. The initial analysis was done by importing transcribed

interviews to notepad and then again imported to open code 4.03 software. Starting from reading several times coding was performed line by line. After checking for similar groups of code were summarized into a category and final themes were created. Categorizing and theming procedures were crosschecked by the advisors and agreed on common categories and themes. The quantitative and qualitative findings were then triangulated.

Ethics approval and consent to participate

This study was approved by the Hawassa University College of medicine, and health sciences research ethics review committee. Written and signed informed consent were obtained from each study participant and head health facilities before the interview. The data collection procedure was anonymous to keep the confidentiality of any information provided by the study participants.

Results

Socio-demographic characteristics of study participants

A total of 235 female health workers participated in this study with a response rate of 97.5%. The mean (± SD) age of study participants was 28.8 (±4.94) years. About 163 (69.4%) of study participants were Hadiya in ethnicity and 178 (75.7%) were protestant religion followers. Concerning their educational status, two third (66%) were degrees and above. One hundred fifty-three (65.1%) of study participants had three and more years of working experience. Nearly three fourth (72.8%) of study participants were married and 142 (60.4%) were working in hospitals. Regarding profession 109(46.4%) of respondents were nurses (**Table 1).**

Table 1. Socio-demographic characteristics of study participants in Hossana town, southern Ethiopia, 2021 (n = 235).

Variables	Categories	Frequency	Percent (%)
Age in years	≤24	40	17.0
	25-34	159	67.7
	≥35	36	15.3
Marital status	Married	171	72.8
	Single	64	27.2
Educational status	Diploma	80	34.0
	Degree and above	155	66.0
Religion	Protestant	178	75.7
	Muslim	13	5.5
	Orthodox	41	17.4
	Catholic	3	1.3
Service area	Hospital	142	60.4
	Health center	93	39.6
Working experience	≤2years	82	34.9
	≥3 years	153	65.1
Professions	Nurse	109	46.4
	Health officer	29	12.3
	Doctors	12	5.1
	Pharmacy	12	5.1
	Laboratory	26	11.1
	Midwifery	34	14.5
	Others*	13	5.5

***=** radiologist, anesthesia

Reproductive and behavioral characteristics

One hundred seventy (72.3%) of study participants had their first sexual intercourse at an age greater than 18 years. Study participants who had a history of multiple sexual partners were 19 (8.1%) and 230 (97.9%) had no history of sexually transmitted disease(STDs). Eighty-nine (37.9%) of respondents were nulliparous women and nearly all (98.8%) of the respondents never smoked (**Table 2**).

Table 2. Reproductive and behavioral characteristics of study participants in hosanna town southern Ethiopia, 2021(n=235).

Variables	Categories	Frequency	Percent (%)
Parity status	Nulli parity	89	37.9
	1-2 child	83	35.3
	≥ 3 child	63	26.8
Age at first sexual intercourse	≤ 18year	65	27.7
	> 18 year	170	72.3
Having multiple sexual partners	Yes	19	8.1
	No	216	91.9
History of STDs	Yes	5	2.1
	No	230	97.9
Smoking status	Yes	3	1.3
	No	232	98.8

Female health worker's knowledge of cervical cancer

One hundred fifty-one (64.3%) of study participants had good knowledge about cervical cancer screening. Regarding symptom-related knowledge, more than half (51.5%) of study participants mentioned risk factors for cervical cancer by respondents were having multiple sexual partners, early sexual intercourse 106 (45.1%), acquiring HPV virus 107 (45.5%), and Cigarette smoking 50 (21.3%). One hundred forty-three (60.9%) of study participants listed vaginal bleeding and foul-smelling vaginal discharge and contact

bleeding accounts (44.7%) and postmenopausal bleeding 49 (20.9%) were the symptoms of cervical cancer.

Knowledge related to cervical cancer screening method were assessed and study participant mentioned pap smear108 (46.0%), HPV DNA test 51(21.7%), VILI 45(19.1%), and only 15 (6.4%) stated the VIA as the screening methods (**Table 3**).

Corresponding to the quantitative finding, key informant interview participants most frequently mentioned female health workers have a low understanding of cervical cancer screening services, as illustrated below:

"….The first hugeness and hardiness of the problem are not understood about the disease and screening service, as I think there is no adequate understanding. However cervical cancer is known as a killer there is no sufficient understanding about prevention methods, risk factors, and availability of screening services, as I think these all may reason for underutilization."[Female, Age:34, Reproductive health specialist]

Another participant reaffirmed the above saying "…*Ok what makes female health workers for not being screened as I tell you above they have no knowledge and awareness and also do not know as the service present in the facility. The first thing they have no specific knowledge about cervical cancer that makes them screen" [Female, Age: 26 midwifery].*

Table 3. Knowledge of risk factors, symptoms, and screening methods among female health workers of Hosanna town southern Ethiopian 2021(n=235).

**Variables**		**No of responded yes**	**Percentage (%)**
Knowledge of Risk factors *	Having multiple sexual partners	121	51.5

	Early sexual intercourse	106	45.1
	Acquiring HPV virus	107	45.5
	Cigarette smoking	50	21.3
Knowledge of symptoms*	Vaginal bleeding	143	60.9
	Foul-smelling vaginal discharge	143	60.9
	Contact bleeding	105	44.7
	Postmenopausal bleeding	49	20.9
Knowledge of screening methods*	Pap smear	108	46.0
	VIA	15	6.4
	VILI	45	19.1
	HPV DNA test	51	21.7

NB. Those with an asterisk (*) were not added up to 100% because of multiple responses

Attitude towards cervical cancer screening

More than half of the respondents (51.5%) had a favorable attitude towards cervical cancer screening. As shown in the Table below around 80.4% of study participants perceived that cervical cancer is the killer cancer in Ethiopia and Cervical cancer screening helps in the prevention of carcinoma of the cervix. Out of the total respondents, 177(75.3%) agree that Cervical cancer screening causes no harm to the clients, and

171(72.8) participants will screen for cervical cancer if the service needs payment. The majority of the participants 188 (80%) agreed that cervical cancer screening tests find changes before it becomes cervical cancer (**Table 4**)

Table 4. Attitude towards cervical cancer screening among female health workers in Hossana town, southern Ethiopia, 2021(n=235)

Variables	Level of agreements	
	Agree Frequency (%)	Disagree Frequency (%)
CC is killer cancer in Ethiopia ?	189(80.4)	46(19.6)
CC screening helps in prevention of carcinoma of cervix ?	188(80)	47(20)
CC screening causes no harm to the clients	177(75.3)	58(24.7)
I will screened for cervical cancer if the service need payment	171(72.8)	64(27.2)
Adult women including you could be acquired cervical cancer	184 (78.3)	51(21.7)
CC screening tests find changes before it becomes cervical cancer	188 (80.0)	47(20.0)
CC screening procedure is embracement	38 (16.2)	197(83.8)
CC screening will you allow male doctors to examine you	155 (66.0)	80(34.0)
If you have cervical cancer do you consult doctors without being scarce	217 (92.3)	18(7.7)

Reasons for not utilizing cervical cancer screening service

The most common reasons mentioned by participants for not being utilized for cervical cancer screening were feeling healthy 119 (54.8%) followed by do not know the place of service 25 (11.5%) and carelessness 24 (11.1%) (**Fig2)**

Figure 2 Main reasons not utilized cervical cancer screening among female health workers in Hossana Town, Southern Ethiopia, 2021.

Corroborating to our quantitative findings, key informant participants articulated that female health workers did not utilize cervical cancer screening services because of a feeling of being healthy; do not know as service is given in their facility, fear of pain, Carelessness of female health workers as illustrated below:

"...a problem is we always think that as we always live healthily. I think the prevalence is increasing currently by understanding these, everyone must be aware as they may acquire the disease I need to tell to all mothers to internalize this idea" (Age: 29, female health workers).

Another key informant indicated that carelessness and knowledge-related factors play important role in getting screened saying

"…. As I think what makes female health worker not to be screened is carelessness, lack of attention and knowledge…." [Female, Age: 26, midwifery working in screening unit].

Around one-third (32.3%) of study, participants mentioned that cervical screening service is not convenient with their regular working time.

In line with this finding, results from a qualitative study showed that female health workers were not convenient with their regular working time. Key informant participants mentioned the major reason that they do not utilize screening services is they are busy and overburdened with their duty, so they have no time to screen. Therefore arranging a

screening on weekend days is necessary. A 26-year-old participant said: "*Indeed female health workers most of the time are busy due to their duty associated with their work. So they have no gate time to screen, therefore arranging to screen on weekend days is necessary to increase cervical cancer screening utilization." [Female, Age: 36, midwifery working in screening unit].*

Cervical cancer screening service utilization

According to the finding of this study, forty-six (19.6%, 95% CI: 14.5%, 24.7%) of the study participants had ever been screened at least once for cervical cancer.

Factors associated with utilization of cervical cancer screening service

Table 5 summarizes a bivariate logistic regression analysis of socio-demographic and other characteristics of study participants that are associated with female health workers' utilization of cervical cancer screening services. Among those variables, age, marital status, educational status, service area, working experience, history of multiple sexual partners, parity, comprehensive knowledge of cervical cancer and its screening method were positively associated with female health worker's utilization of cervical cancer screening services during bivariate analysis (**Table 5**).

Table 5. Multivariable logistic regression analysis on factors associated with utilization of cervical cancer screening among female health workers in Hosanna town, southern Ethiopia, 2021 (n = 235).

Variables	Utilization of cervical cancer screening		COR (95%CI)	AOR (95%CI)
	Yes n (%)	No n (%)		
Age in year				
≤24	4 (10.0)	36 (90.0)	1	1
25-34	30 (18.9)	129 (81.1)	2.09(0.69,6.33)	1.22 (0.34,4.28)
≥35	12 (33.3)	24 (66.7)	4.50(1.21,5.61)	1.38 (0.31,6.33)
Marital status				
Married	40 (23.4)	131(76.6)	2.95(1.18,7.35)	1.64(0.45,6.01)
Single	6 (9.4)	58 (90.6)	1	1
Service area				
Hospital	33 (23.2)	109(76.8)	1.86(0.92,3.76)	1.82 (0.88, 4.05)
Health center	13 (14.0)	80(86.0)	1	1
Parity status				
Nulli parity	9(10.1)	80(89.9)	1	1
1-2 Children	18(21.7)	65(78.3)	2.46(1.04,5.84)	2.15(0.87,5.31)
≥3 children	19(30.2)	44(69.8)	3.84(1.60,9.20)	3.65(1.44,9.21)**
Educational status				
Diploma	24(15.5)	131(84.5)	0.48(0.25,0.93)	0.48 (0.24,0.98)**
Degree and above	22(27.5)	58(72.5)	1	1
Working experience				
≤2 year	9(11.0)	73(89.0)	1	1
≥3 year	37(24.2)	116(75.8)	2.58(1.18,5.67)	1.52(0.57,4.03)
Knowledge of cervical cancer screening				
Good	36(23.8)	115(76.2)	2.32(1.08,4.95)	2.66(1.19,5.95)**
Poor	10(11.9)	74(88.1)	1	1
Had multiple sexual partners				
Yes	8(42.1)	11(57.9)	3.41(1.28,9.04)	3.89(1.38,11.01)**
No	38(17.6)	178(82.4)	1	1

**= significant variables at p value level <0.05   1= reference group

The odds of cervical cancer screening service utilization were 3.65 times [(AOR=3.65; 95% CI: (1.44, 9.21)] higher among female health workers having three or more children as compared to those who were nulliparous female health workers. The odds of cervical cancer screening service utilization among diploma female health workers were 52% less likely [(AOR=0.48; 95% CI: (0.24, 0.98)] when compared with those who were degree and above educational status.

The key informant explained that female health workers with low-level educational status had a low-level understanding so that not utilize the service, saying:

"…When looked at while I provide training, most of the health workers especially those with low educational levels have difficulty with the understanding of cervical cancer screening. Now the problem among health workers a little bite knowledgeable put them in unnecessary confidence resulted in them for the resistant of screening."[Male , Age: 45, gynaecologist].

Concerning knowledge-related factors, the odds of cervical cancer screening service utilization were 2.66 times (AOR=2.66;95% CI: 1.19,5.95) higher among female health workers who had good knowledge of cervical cancer screening, risk factors, and symptoms as compared to who had poor knowledge.

Of nine interviews, six of them said poor knowledge and lack of awareness are the main reason for not utilizing screening services, as illustrated below:

"…..regarding cervical cancer, as I think there is no sufficient knowledge deeply among professionals, so more information and awareness needed."[Female, Age: 35, Public health specialist].

Another participant from the Hospital added her idea:

"… The female health worker most of the time don't know about cervical cancer screening and its knowledge because the service is not provided widely and no media that explain about it…" [Female, Age: 26, midwifery working in screening unit].

Another key informant mentioned even those who had awareness were not properly using cervical cancer screening services, as illustrated below:

"Female health workers did not give attention for cervical cancer screening as a general and they do not know means as there is no anything expose me, they not feel about screening whether they have screened or not screened."[A Female, Age: 28 BSc midwifery].

Similarly, the odds of cervical cancer screening service utilization were 3.89 times (AOR=3.89 95% CI: 1.38, 11.01) higher among female health workers who had a history of multiple sexual partners as compared to those not having multiple sexual partner histories.

Discussion

This study aimed to assess cervical cancer screening utilization and associated factors among female health workers in Hossana town public health facilities. The magnitude of cervical cancer screening utilization was 19.6%. Factors like education level, parity, history of multiple sexual partners, and knowledge of cervical cancer screening were significantly associated with the utilization of cervical cancer screening.

In this study, the finding of cervical cancer screening utilization was 19.6%, which is almost similar to studies that were conducted in Ethiopia (22%), in lower resource settings of Nigeria (20.6%), in Baghdadi (18.8%), in Chennai corporation (18.4%), in Tanzania (15.4%) and other study done in Dar es Salaam, Tanzania (21%)[17-19,23,24]. This

consistency between studies in Ethiopia, Tanzania, and lower resource setting areas was comparable with the current socio-demographic status. However, similar to the study in Baghdadi and Chennai Corporation could be an approachable sample size and study participants.

The finding of this study was lower than the studies done in Saudi Arabia (26.2%), Uganda (75%), Cameroon (41%), and Ibadan, Nigeria (34.6%) [15, 20-22]. This difference might be differences in study settings, sample size, and study participants. Additional justification could be the time of implementing cervical cancer screening practice and the difference in the level of countries' health service coverage. The finding of this study also higher than the studies done in Sidama zone, Southern Ethiopia (11.4%), Arba Minch town, Southern Ethiopia (9.6%), Mekelle town, northern Ethiopia (10.7%), Uttar Pradesh, India (10%), Korea (13%), South-eastern Nigeria (7.2%), rural India (7%), Sokoto, Nigeria (10%) [6-8, 25-29]. This change may be due to time variation, and differences in the study setting majority of study participants were from rural districts and included support staff, but our study participants were mainly female health workers which probably accounted for the observed difference. This suggests the current increase in screening utilization is due to service availability and accessibility in governmental institutions free of charge. It may considerably increase cervical cancer screening utilization.

The most common reasons mentioned by study participants for not being utilized for cervical cancer screening were feeling healthy 119 (54.8%) followed by do not know the place of service 25 (11.5%) and carelessness 24(11.1%). This finding is supported by studies conducted in Korea and Arba Minch town, Southern Ethiopia [6,26]. Additionally,

it can be explained as the fact that when people are feeling healthy they do not bother about preventive services as they have other competing problems. Study participants who do not know the service area of cervical cancer screening further fuel the underutilization of screening services. The finding from a qualitative study in which most participants in in-depth interviews participants indicated a feeling of being healthy supported this, do not know what service is given in their facility, fear of pain, and carelessness as the major barrier to utilizing cervical cancer screening services.

Furthermore, participants described a lack of health educational materials, not having appropriate supplies and logistics, limitation of service to a specific area, service interruption, a distance from the health facility, provider incompetency, and miss-trust and lack of attention by a trained provider and cultural and spiritual factor among female health worker and the unsuitability of environment hindered female health worker from the utilization of cervical cancer screening.

Educational status was one of the significant factors in the utilization of cervical cancer screening services. Diploma female health workers were less likely to utilize cervical cancer screening services when compared with those who had a degree and above educational status. This finding was supported by the studies done in Debremarkos town in Northwest Ethiopia, Wolaita zone, Southern Ethiopia and Nigeria [29-31]. This consistency might be those female health workers who have a degree and above are more educated to have an understanding of the cause, risk factors, prevention mechanism, and screening methods of cervical cancer and as such can demand screening services. Furthermore, education can increase female health workers' access to information from different sources within their educational career and positive effect on

self-efficacy, confidence, and motivation, in search of health interventions for their health including cervical cancer screening utilization. Moreover, interviewees in the qualitative study explained that female health workers with low-level educational status had low-level understanding as a reason for the underutilization of cervical cancer screening.

The odds of cervical cancer screening service utilization were higher among female health workers those having three or more children as compared to those who were nulliparous female health workers. The result of our study is comparable with a study conducted in low resource setting areas of Nigeria, rural India, and Debremarkos Northwest Ethiopia [23, 28,30]. The reason might be explained by these female health workers with three and more children would have experienced repeated exposure to different contact in health facilities (during pregnancy, delivery, postnatal period, immunization, and other health services) might help them to gain information on sexual and reproductive health issues including the benefits of early cervical cancer screening.

The history of multiple sexual partners is also an important predictor of cervical cancer screening utilization. The odds of cervical cancer screening service utilization were higher among female health workers who had a history of multiple sexual partners as compared to those not having multiple sexual partners history. The finding is consistent with a study conducted in the Tigray region, Northern Ethiopia, Debremarkos town, Northwest Ethiopia, and Addis Ababa, Ethiopia [8,30,32]. The possible explanation could be the more sexual partners a woman has, the greater her chances of becoming infected with the human immune-deficient virus and other sexually transmitted diseases including Human Papillomavirus, the most common risk factor for the development of cervical cancer. Therefore, they would have the chance to be infected with the sexually

transmitted disease with its signs and symptoms, which increased health facility visits, and the chance of seeking medical help. Moreover, they might get more health care counseling about cervical cancer screening utilization.

The findings of our study revealed that female health workers' knowledge level has a positive effect on cervical cancer screening utilization. The odds of cervical cancer screening service utilization were higher among female health workers who had good knowledge of cervical cancer screening, risk factors, symptoms, and screening methods as compared to those who had poor knowledge. Similar studies done in Arba Minch town, Southern Ethiopia supported our findings [6]. The qualitative finding also revealed that poor knowledge and lack of awareness contributed to the reasons for not utilizing cervical cancer screening. This consistency might be explained because female health workers with good knowledge about cervical cancer have clear uncertainty about cervical cancer and will have self-initiative to undergo screening and finally increase their awareness about the advantage of knowing about cervical cancer risk factors and benefits of its screening.

The limitation of this study is the fact that since the study design was a cross-sectional study, temporal relations could not be established. In addition to this, since it is a facility-based study and included participants only from selected health institutions, it does not consider women who did not visit and not working in the health facilities. This might affect the representatives of our findings to Hosanna town. The data were self-report by the study participants; thus subject to recall and social desirability bias may affect the result of the study. Regardless of these limitations, our findings have a strength of using the mixed quantitative and qualitative methods allowing for triangulation to confirm findings

and qualitative data to address health facility-related factors, service provider-related factors, female health worker-related factors, and cultural factor inquiry for further explanation of ideas.

Conclusion

The study revealed the magnitude of cervical cancer screening service utilization among female health workers is relatively lower than the Ethiopian national guideline for cervical cancer prevention and control that recommended coverage for the target group. Educational status, parity of respondent, history of multiple sexual partners, and knowledge about cervical cancer were significantly associated with cervical cancer screening utilization. Common reasons given by study participants for not undergoing screening were feeling of health. One-third of study participants revealed that cervical cancer screening time is not convenient.

Moreover, IDIs described that lack of health educational materials, not having appropriate supplies and logistics, limitation of service to a specific area, service interruption, the distance of health facility, provider incompetency, miss-trust and lack of attention by a trained provider, shortage of trained provider and cultural and spiritual factor among female health worker and the unsuitability of environment hindered from the utilization of cervical cancer screening.

Data sharing statement

All relevant data are within the paper and its Supporting Information files.

Funding

This study was not funded.

Author contributions

Conceptualization: Zemzem Jemal and Netsanet Abera

Data curation: Zemzem Jemal

Formal Analysis: Zemzem Jemal and Habtamu Hassen

Methodology: Zemzem Jemal ,Netsanet Abera and Habtamu Hassen

Software: Zemzem Jemal and Habtamu Hassen

Supervision: Netsanet Abera and Nana Chea

Validation: : Zemzem Jemal , Habtamu Hassen, Nana Chea and Netsanet Abera

Writing- original draft: Zemzem Jemal and Habtamu Hassen

Writing-Review and editing: Zemzem Jemal , Habtamu Hassen ,Tsegaab Tesfaye, Nana Chea and Netsanet Abera

All contributed significantly and gave the final approval for the paper to be published; agreed to be accountable for all impacts of the work.

Disclosure statement

The authors declare that there is no conflict of interest in this work.

Acknowledgments

We would like to extend our deepest gratitude to Hawasa University for ethical approval. Our appreciation also goes to the data collectors, supervisors, and study participants.

Ethics approval and consent to participate

Permission to conduct the study was obtained from Hawassa University, College of medicine and health sciences research ethics review committee. Written and signed informed consent were obtained from each study participant and head health facilities before the interview. The data collection procedure was anonymous to keep the confidentiality of any information provided by the study participants.

Abbreviations

AOR, Adjusted Odds ratio; **CC,** Cervical cancer **CI**, Confidence interval; **IDIs**. Indepth interviews; **HPV**, Human Papillomavirus;**STI**, sexually transmitted infections; **SD**, Standard deviation ,**SRS**, Simple random sampling; **H/C**, health center*;* **WUNEMMCSH**, Wachamo University Nigist Elleni Mohammed Memorial comprehensive Specialized Hospital

References

1. Arbyn M, Weiderpass E, Bruni L, de Sanjosé S, Saraiya M, Ferlay J, et al. Estimates of incidence and mortality of cervical cancer in 2018: a worldwide analysis. Lancet Glob Heal. 2020;8(2):e191–203.

2. Barrow A, Onikan A, Nzoputam CI, Ekholuenetale M. Prevalence and determinants of cervical cancer awareness among women of reproductive age: evidence from Benin and Zimbabwe population-based data. Appl Cancer Res. 2020;40(1).

3. WHO/NMH/NMA. UN Joint Global Programme on Cervical Cancer Prevention and Control. The United Nations Global Cervical Cancer Programme 2016.

4. Bruni L, Albero G, Serrano B, Mena M, Go´mez D, Muñoz J, et al. Human Papillomavirus and Related Diseases in Ethiopia. 2017

5. Aranda S, Berkley S, Cowal S, Dybul M, Evans T, Iversen K, Moeti M, Osotimehin B, Peterson S, Piot P, Purandare CN. Ending cervical cancer: A call to action. International Journal of Gynecology & Obstetrics. 2017 Jul;138:4-6.

6. Seyoum T, Yesuf A, Kejela G, Gebremeskel F. Utilization of Cervical Cancer Screening and Associated Factors among Female Health Workers in Governmental Health Institution of Arba Minch Town and Zuria District, Gamo Gofa Zone, Arba Minch, Ethiopia, 2016. Arch Cancer Res. 2017;05(04):4–9.

7. Dulla D, Daka D and Wakgari N. Knowledge about cervical cancer screening and its practice among female health care workers in Southern Ethiopia: a cross-sectional study. Int J Women's Health 2017; 9: 365–372

8. Gebreegziabher M, Asefa NG, Berhe S. Factors Affecting the Practices of Cervical Cancer Screening among Female Nurses at Public Health Institutions in Mekelle Town, Northern Ethiopia, 2014: A Cross-Sectional Study. J Cancer Res. 2016;2016:1–7

9. Ngugi CW BH, Muigai AW, Wanzala P, Mbithi JN. Factors affecting uptake of cervical cancer early detection measures among women in Thika, Kenya. Health Care Women Int. 2012; 33(595–613

10.Brisson M, Kim JJ, Canfell K, Drolet M, Gingras G, Burger EA, et al. Impact of HPV vaccination and cervical screening on cervical cancer elimination : comparative modeling analysis in 78 low-income and lower-middle-income countries. 2020;575–90

11. ACOG. Cervical Cancer Screening 2017 [updated September 2017. https://www.acog.org/Patients/FAQs/Cervical-Cancer-Screening.

12. Federal Democratic Republic of Ethiopia Ministry of Health. Guideline for cervical cancer prevention and control in Ethiopia; 2015.

13. Nwobodo H, Ba-Break M. Analysis of the determinants of low cervical cancer screening uptake among Nigerian women. J Public Health Africa. 2016;6(2):12–9.

14. Hadiya zone health office report 2013 E.C.

15. Obol JH, Lin S, Obwolo MJ, Harrison R, Richmond R. Knowledge, attitudes, and practice of cervical cancer prevention among health workers in rural health centers of Northern Uganda. BMC Cancer. 2021;21(1):1–15.

16. Kress CM, Sharling L, Owen-Smith AA, Desalegn D, Blumberg HM, Goedken J. Knowledge, attitudes, and practices regarding cervical cancer and screening among Ethiopian health care workers. Int J Womens Health. 2015;7:765–78

17. Rashied AM, Yarmook A, Hospital T. Barriers for Uptaking The Pap Smear among Nurse – Midwives in Baghdad City Ahrar M . Rashied , M . Academic Nurse Specialist , Ministry of Health, Al Yarmook. 2014;4(3):1–12.

18. Sudharshini S, Anantharaman V, Chitra A. A cross-sectional study on knowledge, attitude, and practice on cervical cancer and screening among female health care providers of Chennai corporation, 2013. J Acad Med Sci. 2012;2(4):124.

19 Urasa M, Darj E. Knowledge of cervical cancer and screening practices of nurses at a regional hospital in Tanzania. Afr Health Sci. 2011;11(1):48–57.

20. Aldohaian AI, Alshammari SA, Arafah DM. Using the health belief model to assess beliefs and behaviors regarding cervical cancer screening among Saudi women: A cross-sectional observational study 11 Medical and Health Sciences 1117 Public Health and Health Services. BMC Women's Health. 2019;19(1):1–12

21. Tebeu PM, Major AL, Rapiti E, Petignat P, Bouchardy C, Sando Z, et al. The attitude and knowledge of cervical cancer by Cameroonian women; a clinical survey conducted

in Maroua, the capital of Far North Province of Cameroon. Int J Gynecol Cancer. 2008;18(4):761–5.

22. Arulogun OS, Maxwell OO. Perception and utilization of cervical cancer screening services among female nurses in University College Hospital, Ibadan, Nigeria. Pan Afr Med J. 2012;11:69

23. Ifemelumma CC, Anikwe CC, Okorochukwu BC, Onu FA, Obuna JA, Ejikeme BN, et al. Cervical Cancer Screening: Assessment of Perception and Utilization of Services among Health Workers in Low Resource Setting. Int J Reprod Med. 2019;2019:1–8.

24. Kileo NM , Michael D, Neke NM, and Moshiro C, et al. Utilization of cervical cancer screening services and its associated factors among primary school teachers in Ilala Municipality, Dar es Salaam, Tanzania. BMC Health Services Research (2015) 15:552

25. Khanna D, Khargekar N, Budukh A. Knowledge, attitude, and practice about cervical cancer and its screening among community healthcare workers of Varanasi district, Uttar Pradesh, India. J Family Med Prim Care 2019;8:1715-9.

26. Tran NT, Taylor R, Choe SI, Pyo HS, Kim OS, So HC. Knowledge, Attitude and Practice (KAP) Concerning Cervical Cancer and Screening among Rural and Urban Female Healthcare Practitioners in the Democratic People's Republic of Korea. Asian Pac J Cancer Prev. 2011; 12(11):3023–3028.

27. U.W. DOZIE ,ET AL. Determinants of cervical cancer screening uptake among female undergraduates in a tertiary institution in southeastern Nigeria: a cross-sectional study. J PREV MED HYG 2021; 62: E213-E221

28. Shekhar S, Sharma C, Thakur S, Raina N. Cervical cancer screening: knowledge, attitude, and practices among nursing staff in a tertiary level teaching institution of rural India. Asian Pac J Cancer Prev. 2013; 14(6):3641–3645

29. Oche MO, Kaoje AU, Gana G, Ango JT. Cancer of the cervix and cervical screening: current knowledge, attitude, and practices of female health workers in Sokoto, Nigeria. Int J Med Med Sci. 2013;5(4): 184–190

30. Aynalem BY, Anteneh KT, Enyew MM. Utilization of cervical cancer screening and associated factors among women in Debremarkos town, Amhara region, Northwest Ethiopia: Community based cross-sectional study. PLoS One [Internet]. 2020;15(4):1–13.

31. Tekle T, Wolka E, Nega B, Kumma WP, Koyira MM. Knowledge, attitude, and practice towards cervical cancer screening among women and associated factors in hospitals of Wolaita zone, southern Ethiopia. Cancer Manag Res. 2020;12:993–1005

32. Teame H, Addissie A, Ayele W, Hirpa S, Gebremariam A, Gebreheat G, et al. Factors associated with cervical precancerous lesions among women screened for cervical cancer in Addis Ababa, Ethiopia: A case-control study. PLoS One. 2018;13(1):1–13.

Supporting information

S1 Data collection tool (DOC)

S2 SPSS data set (SAV)

S3 Fig 1 (TIFF file)

S4 Fig 2 (TIFF file)

S5 others (pdf)

Figure1

Click here to access/download;Figure;Fig1.tiff

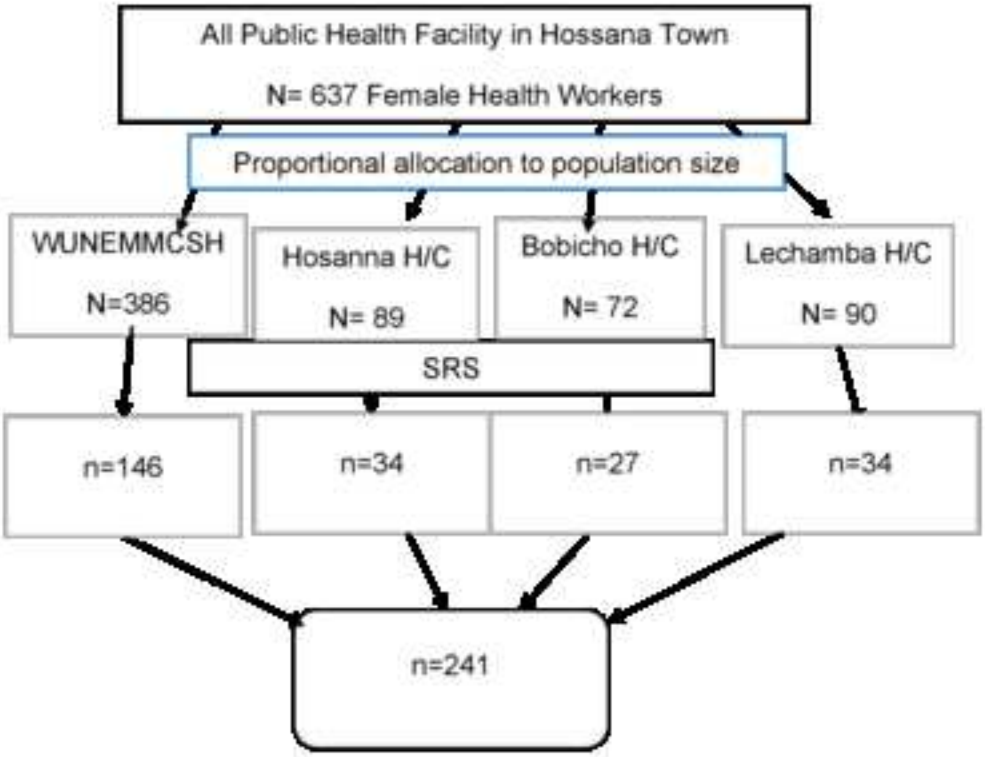

Fig 1: Schematic presentation of the sampling procedure of this study.

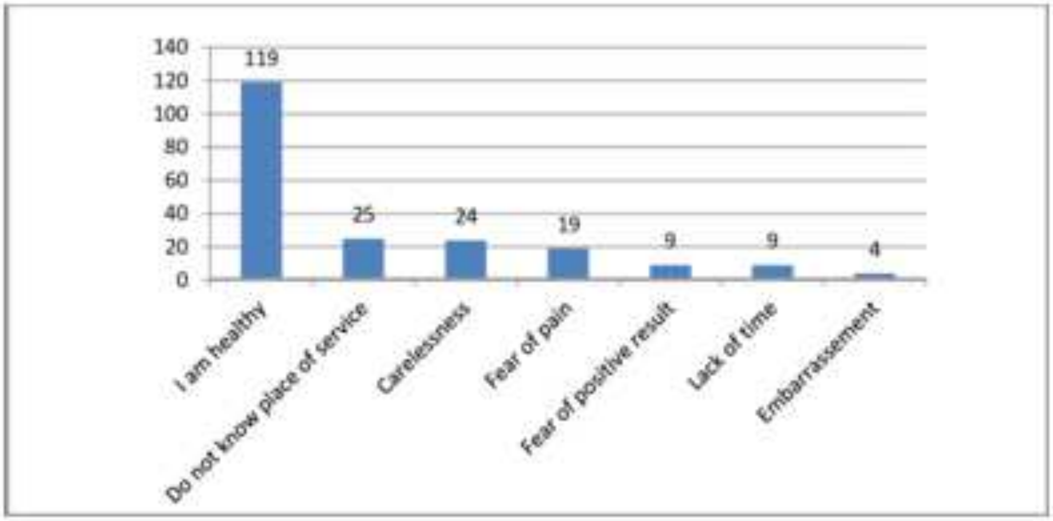

Figure 2 Main reasons not utilized cervical cancer screening among female health workers in Hossana Town, Southern Ethiopia, 2021.

Click here to access/download
Supporting Information
Data collection tool.docx

SPSS files

Click here to access/download
Supporting Information
spss.sav
