# Supplementary material for: Cervical cancer screening utilization and associated factors among female health workers in public health facilities of Hossana town, southern Ethiopia: A mixed method approach
Source: PLoS One. 2023 May 30;18(5):e0286262. doi: 10.1371/journal.pone.0286262 (PMC10228814; doi:10.1371/journal.pone.0286262)
Supplement: Attachment — Submitted filename: Reviewer comments March 2023.docx [file pone.0286262.s005.docx]

**General Comments:** Thank you for the manuscript. Any study on CC in LMIC country is important as efforts are made to control/eliminate CC globally. The manuscript has some merits but needs a lot more to be done to make it publishable.

First, as a general comment, you should get the whole manuscript edited by someone with English as the first language. The current version has significant issues with the use of past and present tenses, the absence of punctuation marks here needed. I have pointed some out below but get this fixed throughout the manuscript.

**Specific comments**

Introduction

1. Line 14: change “portion” to proportion and also you write that the women “had” low CC screening. My concern is with the use of the word “had” in the past tense without reference to a specific year or time. I suggest you write that they have low CC screening…
2. Line 17: health workers role in promotion positive health seeking behaviour including CC screening. The current statement suggest that their role is specific to CC screening
3. Lines 75-77: the sentence does not end in a meaningful way so do rephrase.
4. Lines 82-84: again, another sentence that is confusing. To check and rephrase. It currently state “However, none of these studies have assessed the association between the convenience of screening time and the barrier to utilizing cervical cancer screening services were not qualitatively captured”.
5. Line 84: to state that you aim to determine the “prevalence” in this context is not the most appropriate term. You could say you are determining service utilisation rate or something like that so please rephrase
6. Lines 86-89: this sentence should come just before the study aim.

Methos

7. Lines 100 – 104: rephrase and made the sentence clear and concise.
8. You need to provide more details on the study recruitment process and data collection.
   - How exactly were the HCWs recruited for the quantitative arm?
   - Was this a concurrent mixed method, or sequential or what?
   - How were those HCWs recruited for the interviews for the qualitative arm? Who conducted the interviews?
   - Where, for how long? Etc. etc.
   - Since these were HCWs why were the interviews not conducted in English? What measures were in place to ensure that the translation process, did not affect the content of what was said? Was member checking done after transcription?
   - Could someone who responded to the quantitative survey also be in the qualitative interviews?
   - What informed the sample size for the qualitative interview?
9. Insert the ethical approval reference number.

Results

10. Why was the age ranges done in this manner? Why not start with the youngest age and then create the ranges to be meaningful?
11. Lines 250-251: what does “hugeness and hardiness” mean? If you say this was verbatim, I am wondering what the person said that is translated as hugeness and hardiness. It makes the sentence confusing to the reader. Since the statements after that by the same person sounds clearer, I suggest you delete this first sentence. In fact, the quotations all sound as if the people have low educational level but these are midwives and other level of trained health professionals. I believe the problem is trying to do direct translation from the local dialect to English without giving meaning to the statements. This does not help in this case to make the manuscript read well. Take a look again at your approach to the qualitative data analysis and presentation.
12. Rephrase the sentence at line 296 and 299 about CC service utilisation.
13. Lines 301-304: this whole part needs to be rephrased. The English language use is poor. This really needs to be addressed throughout the manuscript.
14. You repeat to many quotations about the level of CC knowledge, so you must review this and delete some to shorten the manuscript and also make it less boring to the reader.
15. Line 393 has no meaning now. Rephrase because I can’t think through eve to understand what you intend to say
16. Line 395-396: what is lower? Are you still talking about the utilisation rate? If so, be explicit.

Discussion and conclusions

17. These are also full of grammatical errors and these needs to be dealt with.
18. And then it should expand on recommendations which can address the barriers identified in this study.
